# Author Correction: Associative memory by virtual oscillator network based on single spin-torque oscillator

**DOI:** 10.1038/s41598-023-44064-z

**Published:** 2023-10-05

**Authors:** Yusuke Imai, Tomohiro Taniguchi

**Affiliations:** National Institute of Advanced Industrial Science and Technology (AIST), Research Center for Emerging Computing Technologies, Tsukuba, Ibaraki 305-8568 Japan

Correction to: *Scientific Reports* 10.1038/s41598-023-42951-z, Published online 22 September 2023

The original version of this article contained errors in References 49 and 50, which were incorrectly given as:

49. De Wergifosse, S., Chopin, C. & Araujo, F. A. Preprint at https://arXiv.org/quant-ph/2206.13438.

50. Araujo, F. A., Chopin, C. & de Wergifosse, S. Preprint at https://arXiv.org/quant-ph/2206.13596.

The correct references are listed below:

49. De Wergifosse, S., Chopin, C. & Araujo, F. A. arXiv:2206.13438. Preprint at https://arxiv.org/abs/2206.13438.

50. Araujo, F. A., Chopin, C. & de Wergifosse, S. arXiv:2206.13596. Preprint at https://arxiv.org/abs/2206.13596.

The original article has been corrected.

