# Supplementary material for: Associative memory by virtual oscillator network based on single spin-torque oscillator
Source: Sci Rep. 2023 Sep 22;13:15809. doi: 10.1038/s41598-023-42951-z (PMC10517175; doi:10.1038/s41598-023-42951-z)
Supplement: Supplementary file 1 — Supplementary Information. [file 41598_2023_42951_MOESM1_ESM.pdf]

Associative memory by virtual oscillator network based on single spin-torque oscillator

Yusuke Imai[1] **and Tomohiro Taniguchi**[1,*]

[1]National Institute of Advanced Industrial Science and Technology (AIST), Research Center for Emerging Computing Technologies, Tsukuba, Ibaraki 305-8568, Japan
*tomohiro-taniguchi@aist.go.jp

ABSTRACT

This Supplementary Information includes additional results from the numerical simulation, including a table of overlaps in the memorized patterns, another example of the associative memory operation in which there are large overlaps in the memorized patterns, and the dependence of the associative memory operation on noise in the pattern to be recognized.

Degree of overlap between the numerical patterns

Table S1 lists the overlap degrees between the ten memorized patterns, “0”, “1”, $\cdots$, “9”, used in the main text. Remind that we discuss the similarly between the patterns “1” and “7” in the main text. As can be seen in the table, the pattern having the largest overlap with respect to “1” is certainly “7”.

There are other pairs of numbers having large degrees of overlap. For example, the overlap between “3” and “9” is 0.54, which is larger than that between “1” and “7”. Moreover “8” has large overlaps with several other patterns; the overlap with “3”, “5”, “6”, and “9” are 0.73, 0.50, 0.73, and 0.60, respectively. Recall that we concluded in the main text that the associative memory operation fails when the number of the memorized patterns is large and some of the memorized patterns have large overlaps. We also showed that the association becomes accurate when the number of the memorized patterns is reduced or similar patterns are removed from the memorized ones. In the next section, we show that these conclusions are valid even for the memorized pattern “8”.

Associative memory operation for the pattern of “8"

Here, we made a pattern to be recognized by adding noise to the pattern “8” as shown in Fig. S1(a), and then examined the associative memory operation with it. The amplitude of the magnetic field during the association was $\mathscr{H}' = N_{\rm m} \times 0.17$ Oe. First, we used ten memorized patterns, “0”, “1”, $\cdots$, “9”. The finally obtained pattern is shown in Fig. S1(b); it is slightly different from “8” and does not match any of the memorized patterns exactly. This result is similar to the one

Table S 1. Table of overlaps between ten memorized patterns, “0”, “1”, $\cdots$, “9”.

overlap	0	1	2	3	4	5	6	7	8	9
0	1.00	0.37	0.07	0.27	0.13	0.17	0.27	0.30	0.20	0.27
1	0.37	1.00	0.03	0.10	0.03	0.13	0.10	0.47	0.03	0.10
2	0.07	0.03	1.00	0.13	0.20	0.30	0.27	0.17	0.07	0.13
3	0.27	0.10	0.13	1.00	0.13	0.43	0.47	0.30	0.73	0.53
4	0.13	0.03	0.20	0.13	1.00	0.23	0.20	0.10	0.33	0.07
5	0.17	0.13	0.30	0.43	0.23	1.00	0.70	0.07	0.50	0.43
6	0.27	0.10	0.27	0.47	0.20	0.70	1.00	0.10	0.73	0.60
7	0.30	0.47	0.17	0.30	0.10	0.07	0.10	1.00	0.03	0.10
8	0.20	0.03	0.07	0.73	0.33	0.50	0.73	0.03	1.00	0.60
9	0.27	0.10	0.13	0.53	0.07	0.43	0.60	0.10	0.60	1.00

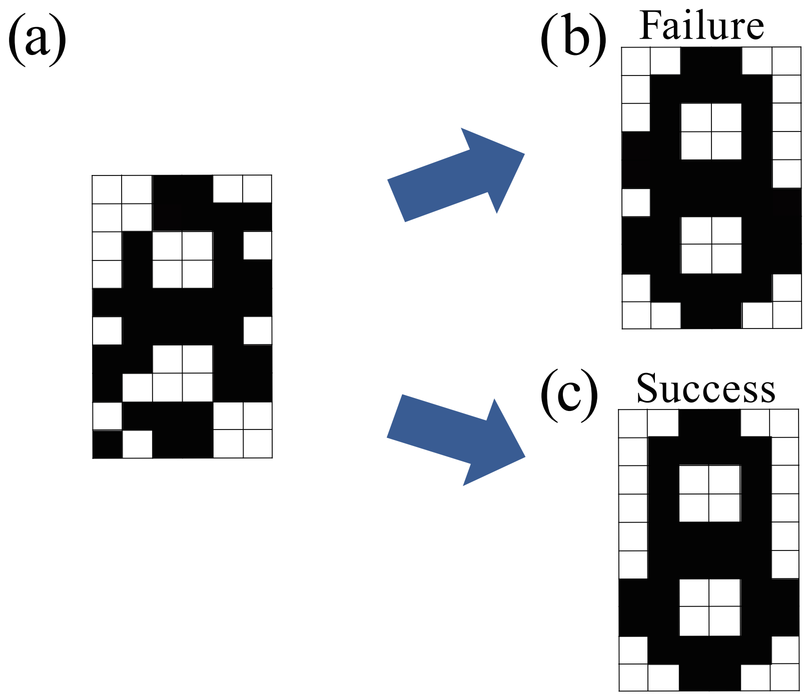

Figure S 1. (a) The pattern to be recognized was obtained by adding noise to the pattern "8". (b) When the memorized patterns included ten patterns, the association of "8" failed. (c) When the memorized patterns included only "3" (or "6") and "8", the association succeeded, even though these patterns have a large overlap.

shown in the main text, where the pattern to be recognized was similar to "1" but had noise in it, and the finally obtained pattern did not correspond to any of the memorized patterns when the number of the memorized patterns was ten. Next, we examined the association by removing some of the memorized patterns. For example, when we used only "3" and "8" as the memorized patterns, the finally obtained pattern eventually becomes "8", as shown in Fig. S1(c); thus, the association succeeded. Moreover, the association succeeded even when we used "6" instead of "3". These results support the conclusions in the main text.

Dependence of associative memory operation on noise

Here, we investigate the dependence of the accuracy of the associative memory operation on the number of noisy pixels in the pattern. Before showing the results, however, we should define what we mean by noise (or noisy pixel) and accuracy.

Noise is defined as swapping black and white colors in a memorized pattern to make a new pattern to be recognized. For example, the pattern to be recognized in Fig. S1(a) is obtained by swapping the colors of 8 pixels from the pattern in Fig. S1(c); therefore, there are 8 noisy pixels in this case. If the virtual oscillator network can associate the pattern in Fig. S1(a) with that Fig. S1(c), we say it is an accurate association. If the finally obtained pattern still includes some noise, as in the case of Fig. S1(b), it is classified as an inaccurate association.

Here, one might imagine other definitions of accuracy. As mentioned above, we prepared the patterns to be recognized by adding a noise to one of the memorized patterns. Let us call the pattern to be recognized R and the original memorized pattern A. If the amount of noisy pixel is large, R might become more similar to one of the other memorized patterns, call it pattern B. Mathematically, this means that the overlap between R and B is larger than that between R and A, even though the pattern R is generated from the pattern A. In this case, the finally associated pattern might be B. Similarly, even when the overlap between R and A is the larger than those between R and any of the other memorized patterns, the finally obtained pattern might become one of the other memorized patterns. We regarded such associations to be inaccurate, even though the finally obtained pattern is amongst the memorized ones. This is because noise reduction (or pattern recovery) remains a challenging aspect of the associative memory operation, so we aimed to associate R with the pattern it is derived from, i.e., A.

With these definitions of noise (noisy pixel) and accuracy in hand, let us evaluate the accuracy as a function of the number of noisy pixels. Here, let us consider associating "1" from three memorized patterns, "0", "1", and "2", as in the case studied in the main text. As mentioned in the Methods, the maximum number of noisy pixels is $N/2$, where N is the number of the pixels, 60 in the present study.

Figure S2(a) shows an example of the association in which the pattern to be recognized was generated by adding 30 noisy

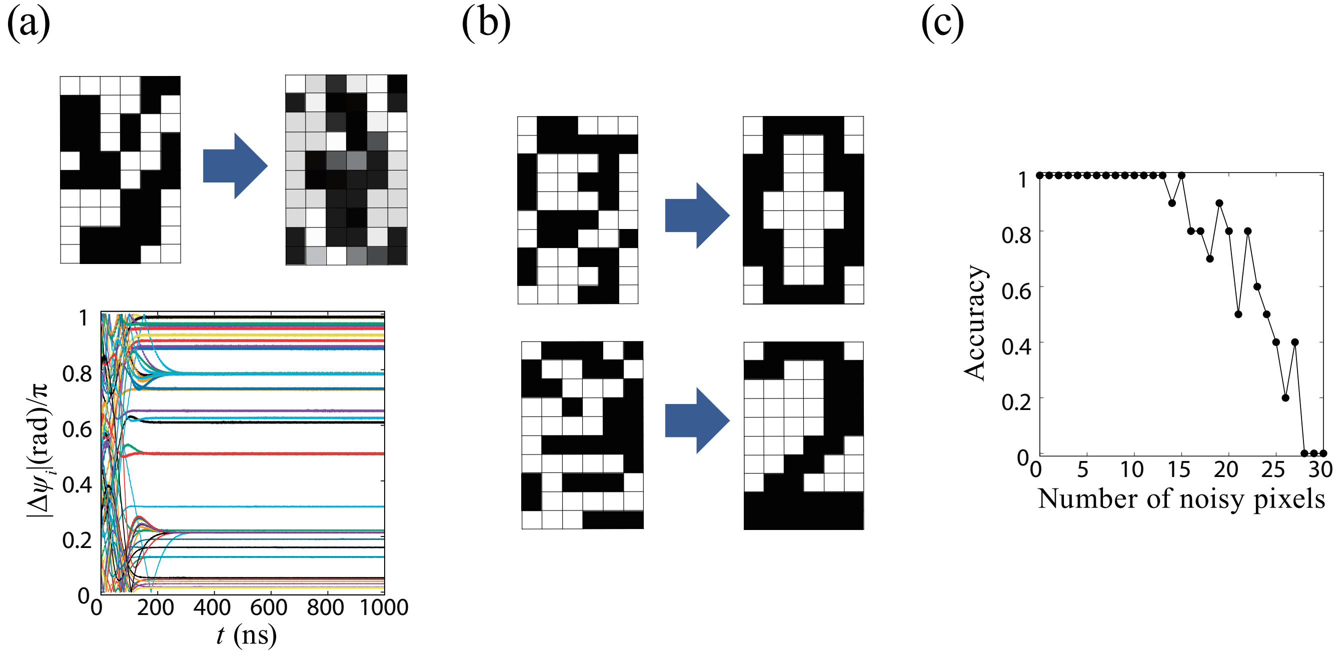

Figure S 2. (a) Example of an inaccurate association. The top left and right patterns are the pattern to be recognized and the final pattern obtained after the association. In this case, the final pattern does not match any of the memorized ones (“0”, “1”, and “2”). In particular, some of its pixels are colored neither black nor white. This means that some phase differences did not saturate to π or 0, as shown in the bottom figure. (b) Other examples of inaccurate associations. In these cases, the finally associated patterns are “0” and “2”, even though the patterns to be recognized were generated by adding noise to “1”. (c) Dependence of accuracy of the associative memory operation on the number of noisy pixels.

pixels to pattern “1”. In this case, the finally associated pattern is not “1”. Furthermore, some of the pixels are neither black nor white. This means that the phase differences of some parts did not saturate to either π or 0 [see Fig. S2(a)]. Such a gray pattern is obtained when the number of noisy pixels is relatively large; in contrast, when the number of noisy pixels is relatively small, the finally obtained pattern is black-and-white even if the association fails, as shown in Fig. S1(b).

Figure S2(b) shows other examples for which the patterns to be recognized were obtained by adding 30 noisy pixels to “1”. The finally associated patterns are other memorized patterns, “0” and “2”. Here, the overlaps between the patterns to be recognized and “0” and “2” are relatively small; the overlaps between the patterns to be recognized and the finally obtained patterns were 0.1. Even for such a small overlap, the association became inaccurate because the overlaps between the patterns to be recognized and “1” are zero. Moreover, the finally obtained pattern has the largest overlap with the pattern to be recognized. For example, in the top example in Fig. S2(b), the overlaps between the pattern to be recognized and the memorized patterns are 0.1 for “0”, 0 for “1”, and 0.03 for “2”. Similarly, in the bottom example in Fig. S2(b), the overlaps are 0.03 for “0”, 0 for “1”, and 0.1 for “2”. Therefore, the finally associated pattern is the one with the largest overlap. According to the definition of accuracy above, however, these examples should be regarded as inaccurate associations.

Figure S2(c) summarizes the accuracy as a function of the number of noisy pixels. Here, we quantified the accuracy as follows. We prepared ten ($N_e = 10$) patterns to be recognized for a certain number of noisy pixels, where noisy pixel was added randomly to the ten patterns. For example, the patterns shown in Figs. S2(a) and S2(b) are examples of such patterns with 30 noisy pixels. The accuracy in Fig. S2(c) is the number of the correctly associated patterns divided by $N_e = 10$. Therefore, the accuracy is 1 when the association succeeds for $N_e = 10$ prepared patterns to be recognized, while the accuracy is zero when the association fails for all prepared patterns. We used “0”, “1”, and “2” as the memorized patterns and generated the patterns to be recognized by adding noisy pixels to pattern “1”. The accuracy was 1 for relatively small number of noisy pixels. It deviated from 1 when the number of noisy pixels was close to half of the maximum value, i.e., the association was inaccurate when the number of noisy pixels was approximately larger than $N/4$. The accuracy was zero when the number of noisy pixels was close to the maximum number, $N/2$, because the overlap between the pattern to be recognized and the original pattern becomes approximately zero.
